# Distribution models and species discovery: the story of a new *Solanum* species from the Peruvian Andes

**DOI:** 10.3897/phytokeys.31.6312

**Published:** 2013-12-16

**Authors:** Tiina Särkinen, Paúl Gonzáles, Sandra Knapp

**Affiliations:** 1 Royal Botanic Garden Edinburgh, 20A Inverleith Row, EH3 5LR Edinburgh, United Kingdom; 2 Plants Division, The Natural History Museum, Cromwell Rd, SW7 5BD London, United Kingdom; 3 Laboratorio de Florística, Departamento de Dicotiledóneas, Museo de Historia Natural - Universidad Nacional Mayor de San Marcos, Avenida Arenales 1256, Apartado Postal 14-0434, Lima, Peru

**Keywords:** Tropical Andes, Solanaceae, species distribution modelling, Peru, endemism, rare species, Morelloid Clade, *Solanum* section *Solanum*

## Abstract

A new species of *Solanum* sect. *Solanum* from Peru is described here. *Solanum pseudoamericanum* Särkinen, Gonzáles & S.Knapp **sp. nov.** is a member of the Morelloid clade of *Solanum*, and is characterized by the combination of mostly forked inflorescences, flowers with small stamens 2.5 mm long including the filament, and strongly exerted styles with capitate stigmas. The species was first thought to be restricted to the seasonally dry tropical forests of southern Peru along the dry valleys of Río Pampas and Río Apurímac. Results from species distribution modelling (SDM) analysis with climatic predictors identified further potential suitable habitat areas in northern and central Peru. These areas were visited during field work in 2013. A total of 17 new populations across the predicted distribution were discovered using the model-based sampling method, and five further collections were identified amongst herbarium loans. Although still endemic to Peru, *Solanum pseudoamericanum* is now known from across northern, central and southern Peru. Our study demonstrates the usefulness of SDM for predicting new occurrences of rare plants, especially in the Andes where collection densities are still low in many areas and where many new species remain to be discovered.

## Abstract (es)

Se describe una nueva especie de *Solanum* sección *Solanum* endémica del Perú. *Solanum pseudoamericanum* Särkinen, P. Gonzáles & S.Knapp **sp. nov.**, es un miembro del clado Morelloide de *Solanum*. Se caracteriza por presentar inflorescencias bifurcadas, flores con estambres pequeños de 2.5 mm de largo incluyendo el filamento y estilos muy exertos con estigma capitado. En un principio se pensó que la especie estaba restringida a los bosques tropicales estacionalmente secos del sur del Perú, a lo largo de los valles secos de los ríos Pampas y Apurímac. Los resultados del análisis de los modelos de distribución de especies (SDM) con predictores climáticos identificaron posibles áreas con hábitat adecuado en el norte y centro del Perú. Durante el trabajo de campo en el 2013 estas áreas fueron visitadas. Se descubrieron 17 nuevas poblaciones ubicadas dentro de la distribución predicha, y aunque sigue siendo endémica en el Perú, *Solanum pseudoamericanum* ahora es conocida en todo el norte, centro y sur de Perú. Nuestro estudio demuestra la utilidad de SDM para predecir nuevos registros de plantas raras, sobre todo en los Andes, donde la cantidad de colecciones sigue siendo baja en muchos areas y donde muchas especies nuevas aún no se han descubierto.

## Introduction

The tropical Andean hotspot is one of the most species rich but data poor areas of the world (Swenson et al. 2012). The area is estimated to contain c. 45,000 vascular plant species (Orme et al. 2005; Mittermeier et al. 2005; Olson and Dinerstein 2002), slightly more than the entire flora of Brazil catalogued thus far (http://floradobrasil.jbrj.gov.br , accessed on Sept 2013). At the meeting point between the northern and central Andes, Peru alone hosts 19,232 plant species, of which 5,581 (29%) are endemic (Brako and Zarucchi 1998; León et al. 2006; Jørgensen et al. 2011).

Many more species remain to be discovered, however, especially in Peru and Ecuador, where the number of new discoveries per year shows no sign of diminishing (Joppa et al. 2011). In fact, estimates based on taxonomic effort over time project that up to 6,400 species of vascular plants remain to be discovered in the area (Joppa et al. 2011). The high number of undescribed species is not surprising considering the generally low collection density of vascular plants in the Andes (Distler et al. 2009). New discoveries continue to be made even in taxonomically better-known groups such as birds (Hosner et al. 2013; Seeholzer et al. 2012), lizards (Venegas et al. 2013), and mammals (Jiménez et al. 2013; Helgen et al. 2013). It is clear that further collections are needed to completely describe the area’s biodiversity and to fully understand species distributions in the Andes.

In an effort to speed up the process of cataloguing species diversity and recording accurate distributions, an approach referred to as Model-Based Sampling (MBS) has been developed (Guisan et al. 2006). MBS uses Species Distribution Models (SDM) to create maps of potentially suitable habitat areas for poorly known and/or yet undescribed species. Areas with similar environmental conditions that are identified in the modelled maps are then targeted during field work. Although the power of MBS has been shown in previous studies across geographic regions and taxonomic groups (Raxworthy et al. 2003; Le Lay et al. 2010; Boetsch et al. 2003; Williams et al. 2009; Edwards et al. 2005; Guisan et al. 2006; Bourg et al. 2005), more case studies are needed to demonstrate that MBS analyses prior to field work can aid in species description and discovery, especially in tropical areas where collection densities remain low.

Here we present a case study of MBS from *Solanum*, one of the most species rich vascular plant genera in the Andes. In Peru alone *Solanum* includes 299 species, of which 102 are endemic (Knapp et al. 2006; Jørgensen et al. 2011), and many new species continue to be discovered (e.g., Stern and Bohs 2010; Knapp 2010a, b; Farrugia and Bohs 2010). Currently we are in the process of revising the Morelloid clade, one of the largest groups of *Solanum* in the Andes lacking a taxonomic monograph. The Morelloid clade consists of five morphological sections (sections *Solanum*, *Campanulisolanum*, *Parasolanum*, *Chamasarachidium*, and *Episarcophyllum*), and includes c. 68 species of which c. 58 are endemic to the tropical Andes (Bohs 2005). Several new species have been identified during the taxonomic study and are awaiting formal description. Here we describe one of these, *Solanum pseudoamericanum* sp. nov. Särkinen, Gonzáles & S.Knapp, originally known only from four collections from southern Peru. We use MBS to perdict areas containing new populations and confirm the validity of the approach by locating the plants through targeted field work.

## Methods

### Species description

We examined 26 herbarium specimens in the herbaria listed in the text. These were combined with our field observations from Peru in the identification and description of the new taxon (see Taxonomy below). All specimens are cited in the text and full data is provided in the supplemental file and on Solanaceae Source (www.solanaceaesource.org ). We included all specimens examined in the model-based analysis.

### Model-based sampling (MBS)

Following the MBS approach by Guisan et al. (2006), we used a SDM method to identify potentially suitable habitat areas for *Solanum pseudoamericanum*. We chose the machine learning algorithm MAXENT version 3.3.3e (Phillips et al. 2006) for developing the habitat suitability maps. MAXENT uses the principle of maximum entropy to discriminate the range of environments associated with species occurrences from the range of environmental conditions present across the landscape and finds the smoothest climatic envelope that describes the presence points. MAXENT is considered one of the most reliable methods when working with a small number of potentially biased occurrence records (Hernandez et al. 2006; Pearson et al. 2007; Wisz et al. 2008; Elith et al. 2006), and has been found to outperform other SDM methods in complex but poorly collected areas (Hernandez et al. 2008). MAXENT requires presence points only and can hence be used for modelling poorly known species for which reliable absence records cannot be derived.

### Identifying potential new populations

We first ran MAXENT based on the four observed collections from 2012 from southern Peru to identify potential suitable habitat areas for the target species (Model 1). The model was run with default settings (allowing for transformations of the covariates with the default thresholds for conversion, removing duplicate presence records, maximum number of background points = 10,000, maximum number of iterations = 500; convergence threshold = 0.00001; fit regulization parameter = 1; default prevalence = 0.5). To evaluate model performance, we ran it with cross-validation, where the occurrence data is randomly split into two equal-sized groups and one of them is then used for creating the model whilst the other is used for validating the model. We chose cross-validation approach because it uses all of the data for validation, unlike a single training/test split, and is hence more suitable when working with small numbers of occurrence points across a complex landscape (Hernandez et al. 2008).

The model was run with 11 bioclimatic variables at 30 arc second spatial resolution (c. 1 km^2^) (Hijmans et al. 2005; http://www.worldclim.org ). Variables used in models as predictors should optimally be independent. In order to avoid colinearity between the climatic variables, we tested for correlations between all the 19 BioClim and the digital elevation model (SRTM) using Principal Component Analyses, and excluded nine variables that were found to be highly correlated with Pearson correlations coefficients of 0.75 or higher (Table 1). The remaining 11 variables included layers describing the seasonality of the habitat (Mean Diurnal Range, Temperature Seasonality, Temperature Annual Range and Precipitation Seasonality) and precipitation and water availability (Precipitation of Wettest Quarter, Precipitation of Driest Quarter, Precipitation of Warmest Quarter, Max Temperature of Warmest Month, Mean Temperature of Driest Quarter) (Table 1). The model was trained using southern Peru alone (-76, -70, -15.2, -12), and the results of the training were then projected over the whole of Peru (-81.6, -68.0, -18.5, 0). This approach of limiting the model training extent avoids model overfitting which leads to underestimation of species’ distribution areas (Barve et al. 2011).

**Table 1.** Principal components analysis (PCA) results of the climatic variables (http://www.worldclim.org ) used to generate distribution models for *Solanum pseudoamericanum*. Variables with Pearson correlation coefficients equal or greater than 0.75 were removed.

	**PC1**	**PC2**
**Cumulative variation explained**	47%	70%
**BIOCLIM VARIABLES USED**	**Eigenvectors**	
Mean Diurnal Range (BIO2)	0.085	-0.117
Isothermality (BIO3)	-0.095	-0.001
Temperature Seasonality (BIO4)	0.044	0.175
Max Temperature of Warmest Month (BIO5)	-0.277	0.121
Min Temperature of Coldest Month (BIO6)	-0.304	0.160
Temperature Annual Range (BIO7)	0.097	-0.094
Mean Temperature of Driest Quarter (BIO9)	-0.300	0.148
Precipitation Seasonality (BIO15)	0.070	0.346
Precipitation of Wettest Quarter (BIO16)	-0.224	-0.254
Precipitation of Driest Quarter (BIO17)	-0.235	-0.278
Precipitation of Warmest Quarter (BIO18)	-0.109	-0.253

The Model 1 output was ground-truthed with additional field work. To target areas where potential new populations of *Solanum pseudoamericanum* could be encountered, we chose to interpret the Model 1 cumulative output. We chose not to use a threshold approach, where the prediction is divided into a binary map of presence or absence, due to the fact that only four records were used for building the model and hence using a threshold approach would discard valuable data. The cumulative output indicates relative suitability, not probability, of occurrence with values ranging from 0 to 100. Grid cell values are calculated as the sum of the cells with equal or lower probability, multiplied by 100 to give a percentage (Phillips et al. 2006). All areas identified in the Model 1 with a relative suitability of more than 40% were considered as high priority areas for ground-truthing during the second field season in April-June 2013. Further occurrence records for the new species were identified through herbarium visits and loans. Local Peruvian herbaria were visited in Arequipa (HUSA), Lima (MOL, USM), Trujillo (HUT) and Cajamarca (CPUN), and loans from several international herbaria were examined (F, MO, US, S, NY).

### Potential distribution map

We ran a second model after the second field season, where all new localities identified through field work and herbarium visits and loans were included. Model 2 was run using a total of 26 records, of which four were from our first field trip in 2012, 17 were from our second field trip in 2013, and five from herbarium records (SI File 1 Occurrence data). The same 11 climatic predictors and MAXENT parameters were used as in Model 1 (see above). The model was trained using Peru as the study extent, and results were projected to an area that covered the whole of Ecuador and northern Bolivia (-81.0, 65.6, -19.5, 0). A final potential distribution map for *Solanum pseudoamericanum* was produced based on the cumulative output of Model 2, where all areas with relative suitability above 0.4 (logistic output) were considered as potential areas of occurrence for the species.

## Results

We evaluated the relative success of our SDM model predictions based on the mean area under curve (AUC) values of the receiver operating characteristic (ROC) curve of the cross-validation replicates. AUC values close to 1 indicate optimal performance, whilst values close to 0.5 indicate performance equal to random. Both models yielded AUC values > 0.98 indicating good model performance (Table 2). The two most important climatic variables included in Models 1 and 2 were precipitation of the driest quarter, temperature seasonality, and minimum temperature of the coldest month based on jacknife analyses of variable importance. Other important variables included isothermality (mean diurnal range coupled with annual temperature range) and maximum temperature of warmest month.

**Table 2.** Model performance values for the two models run to detect suitable habitat areas for *Solanum pseudoamericanum*.<br/>

**Model**	**No. of records**	**AUC score (mean)**	**Standard deviation**
Model 1	4	0.987	0.009
Model 2	26	0.984	0.014

**Figure 1.**
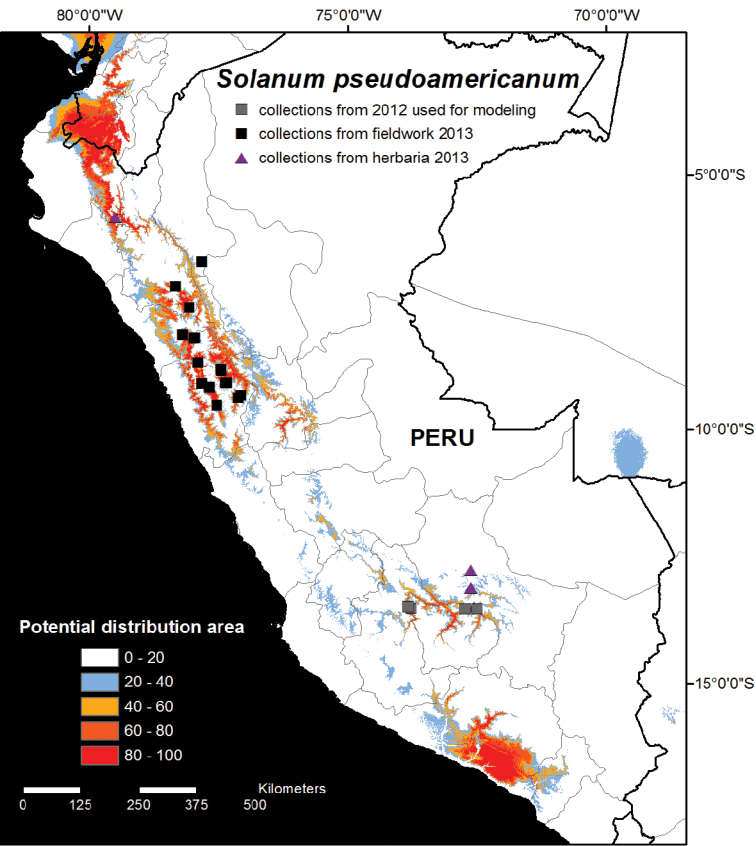
Potential habitat distribution map of *Solanum pseudoamericanum*. The potential habitat areas reflect the cumulative output of the MAXENT model produced using 11 climatic variables with the original four collection localities from 2012 from southern Peru shown as grey squares on the map (see Methods for details). Areas identified as highly suitable (above 40% cumulative probability) in central and northern Peru were visited in 2013 during the second field season, and 17 new collection localities were found as a result (black squares). Five additional collections were identified amongst herbarium loans (purple triangles).

The results of Model 1, using only the first four records from 2012, showed highly suitable climatic conditions in northern and central Peru in the Departments of Cajamarca, La Libertad, Ancash and Huánuco, as well as in northernmost Piura and Loja, El Oro and Azuay provinces of Ecuador (Fig. 1). The core suitable areas were visited in Cajamarca, La Libertad, and Ancash during the second field season, and 17 new populations were identified (Fig. 1). Five specimens were identified amongst herbarium loans from NY and MO, collected from Piura and Cusco (Fig. 1; Appendix). Surprisingly, no collections of *Solanum pseudoamericanum* were found in local herbaria in Peru. Model 1 also identified highly suitable habitat areas in southern Moquegua and Arequipa (Fig. 1). These areas were visited in 2012 during our first field season and whilst many *Solanum* collections were made, no specimens of *Solanum pseudoamericanum* were observed.

**Figure 2.**
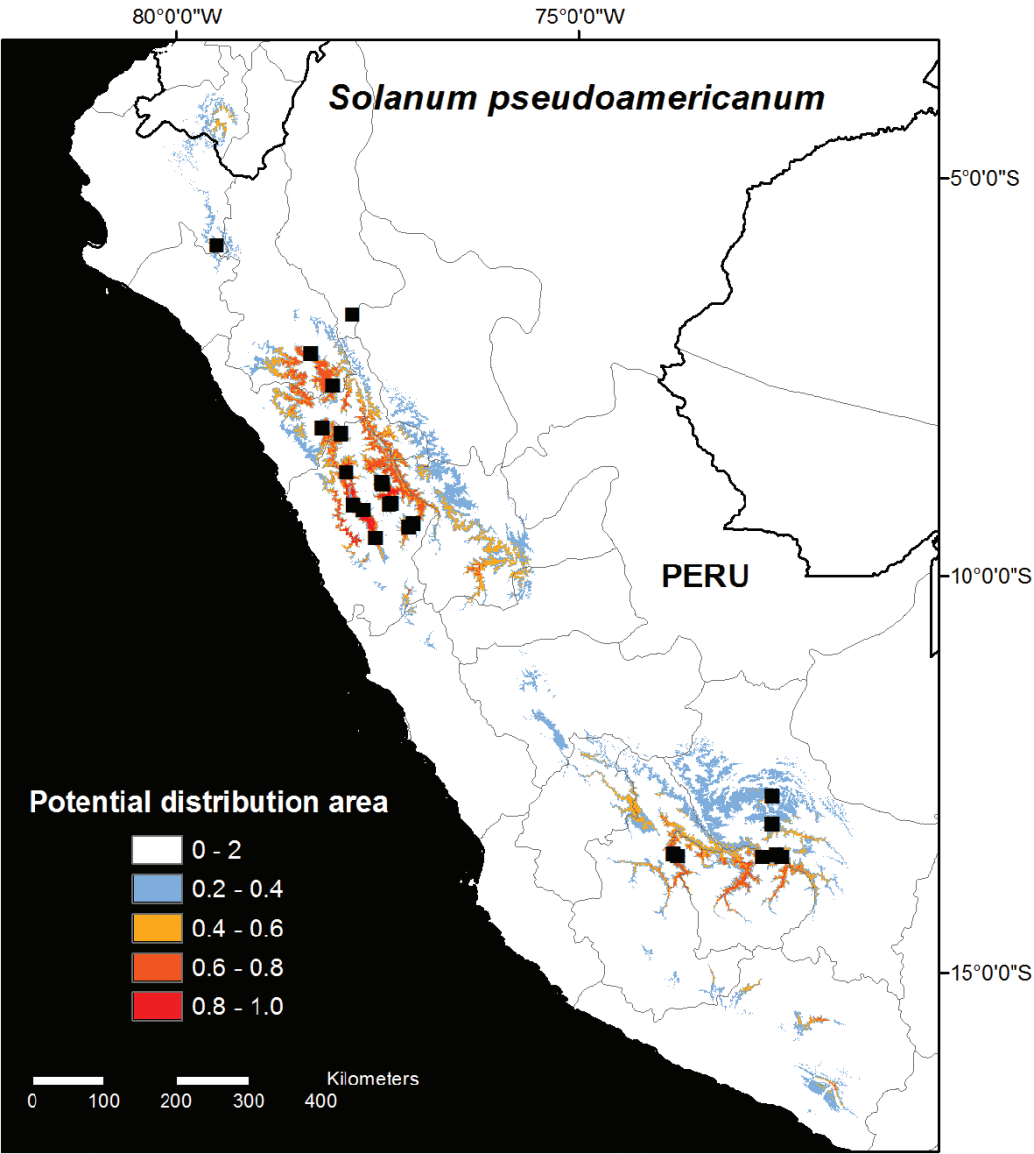
Distribution map of *Solanum pseudoamericanum*. The potential habitat areas reflect the logistic output of the MAXENT model produced using 11 climatic variables with all current known occurrence records (N=26; Model 2).

Model 2 was run with all collection data from 2012 and 2013, including all herbarium collections (Fig. 2). The Model 2 prediction was generally similar to Model 1, but Model 2 predicted a smaller range size to the species where no suitable habitat areas are predicted to occur outside Peru except in Loja, Ecuador, and only small areas of likely habitat area are found in Arequipa (Fig. 2). The smaller predicted distribution area in Model 2 was despite the fact that the results were projected over larger area covering both Ecuador and northernmost Bolivia. Areas identified in Model 2 as likely habitat areas but which remain unconfirmed include southwest San Martín, Huánuco, northern Pasco, Huancavelica, Junín, and Arequipa, as well as Loja, Ecuador (Fig. 2).

## Discussion

### Can SDM help in finding rare species?

Previous studies have clearly demonstrated how the use of SDM can dramatically increase detection rates of rare species in the field (Guisan et al. 2006; Raxworthy et al. 2003; Edwards et al. 2005; Boetsch et al. 2003). Our case study adds to this list of studies where records of rare species are used to locate new populations via SDM. Once new populations are found, new models are reiteratively run to enhance the distribution models (La Ley et al. 2010). Our example demonstrates that the MBS approach can be used even in more complex and poorly collected areas such as the Andes, and can greatly help in increasing our knowledge of species distribution patterns in highly diverse systems. It is clear from the continuing rates of species discovery in plants (Joppa et al. 2011) as well as in mammals and birds in the tropical Andes (Hosner et al. 2013; Seeholzer et al. 2012; Jiménez et al. 2013; Helgen et al. 2013) that tools such as SDM should be used to predict diversity patterns from the existing sparse data.

### Modelled versus observed distribution maps

Here we describe a new species and provide both an observed distribution map as well as a modelled distribution range for the species. With increasing ease of SDM through publicly supported online portals such as BioVel (http://www.biovel.eu ), the tools are now available for non-specialists to analyse models prior to species publication. Generally, SDMs are still created by GIS specialists rather than taxonomic specialists, but the availability of online portals will hopefully increase the use of SDM amongst taxonomists who are best informed to run such models because of their expert knowledge of species’ ecology.

Modelled distribution maps have large benefits over observed distribution maps. Modelled maps, although still incomplete, can be argued to provide a more realistic picture of the actual species’ distribution area. This is because modelled maps are less biased by collection densities, and although nowhere near complete, provide a step towards representing species distributions in a more realistic manner. Such maps will also aid in targeting field collecting efforts and provide additional information for planning conservation areas compared to traditional maps.

Whilst advocating the publication of modelled distributions for new species, we fully acknowledge that species distributions are not guided by simple factors such as climate alone. Many factors govern range size, including dispersal limitation, competitive exclusion, habitat destruction, urbanisation and agriculture, as well as species interactions. These complex factors are often dismissed in simplistic SDMs where only bioclimatic predictors are included. Simple SDMs can, however, be used as a starting point for evaluating rare species (e.g., Simon et al. 2011). Firstly, SDMs can be used to establish whether species are truly restricted in their distributions by reducing sampling artefacts such as those presented here. Secondly, simple SDMs can be used as null models to examine whether bioclimatic factors restrict species’ distributions or whether other factors, such as dispersal limitation or habitat destruction, are likely at play.

### Against the odds?

In the case of *Solanum pseudoamericanum*, the MBS approach helped us to extent the range size of the newly described species, changing our view of the target taxon from a narrow endemic species restricted to only two river valleys in southern Peru to a relatively widespread species that is distributed across Peru. The large increase in the actual observed distribution range of the new species demonstrates not only how poorly collected the Peruvian Andes is for vascular plants, but also how MBS can work with extremely low number of collection records across a complex landscape. This extension of the observed occurrence area of the newly described species was despite the relatively large model training area that was used, where the whole of Peru was considered. The use of relatively large training areas in model training leads to model overfitting and underprediction of distribution areas (Barve et al. 2011), which in our case means that there is likely to be a bias towards underestimating the true potential habitat area of *Solanum pseudoamericanum*. But is our case study an exception?

Our null hypothesis was that MBS approach cannot be used in such a highly variable landscape as the Andes with as few records as we had available. Our expectations were low for two reasons. Firstly, the climate data available for the Andes through WorldClim suffers from high uncertainty because only a few weather stations were used to interpolate the data (Hijmans et al. 2005). Hence, we expected that the climatic data might not be adequate to produce good models for Andean species. Secondly, we expected poor model performance due to the low number of records used. Although some algorithms, such as MAXENT which was used here, have been found to be less sensitive to small sample sizes than other methods, they still require generally more than 30 records to obtain accurate results (Wisz et al. 2008; Kadmon et al. 2003).

Results from our case study indicate that both assumptions might not be correct. The high AUC scores shown by our models indicate that informative models can be run with as little data as used here and with climate predictors alone. The climate data appears to be of high enough quality to reveal broad patterns that can be used to identify suitable habitats across poorly explored regions. Variation in climate, and the associated elevational gradients, seem to explain large parts of plant distribution patterns in the Andes (Killeen et al. 2007), and hence such simple bioclimatic models can perform well. This is in contrast to lowland Amazonia where climatic variation, as well as elevational gradients, are much reduced and where the importance of soil in explaining diversity patterns has been highlighted (Hoorn et al. 2010; Higgins et al. 2011; Tuomisto et al. 2003).

Another question is the minimum number of occurrence records required for building accurate distribution models. While it is well established that more data produce better, more accurate models (Wisz et al. 2008; Kadmon et al. 2003), the question remains how little is enough to produce an informative SDM? The good news based on our case study is that the actual number of records might not be the only thing that matters. As exemplified by *Solanum pseudoamericanum*, a small number of records can be enough to characterize the climatic niche of a species, given that the few records available adequately describe the environment that the species occupies. In other words, it is not only the pure number of records but the information content that the locality points provide that matters (Kadmon et al. 2003). Optimally, occurrence records should be spatially spread and represent the environmental extremes that the species occupies (Syfert et al. 2013). Because the information content of the occurrence points is often hard to know a priori, our case study demonstrates the value of running preliminary SDM analyses even when only a handful of records are available. Caution has to be given to how SDM analyses are run with limited data, and care should be especially be given to interpreting AUC values which can be inflated due to small number of records (Wisz et al. 2008), especially when sampling bias is present (Raes and ter Steege 2006). Despite this caution, our results presented here are encouraging and we see SDMs as a tool that can offer much needed help in our efforts to describe diversity in poorly explored areas such as the Andes.

## Taxonomic treatment

The new species described here belongs to *Solanum* section *Solanum* within the Morelloid clade (sensu Bohs 2005) of *Solanum*. The section contains species that are unarmed shrubs and herbs to 2 m tall with simple or branched glandular or eglandular trichomes, simple to many times branched internodal inflorescences and small berries with multiple small seeds and usually containing stone cells.

### Solanum pseudoamericanum

Särkinen, P.Gonzáles & S.Knapp sp. nov.

urn:lsid:ipni.org:names:77134672-1

http://species-id.net/wiki/Solanum_pseudoamericanum

Figs 2 [Fig F3] –4

#### Diagnosis.

Like *Solanum americanum* L. but differing in branched inflorescences with flowers spaced along the rachis (not umbellate), rounded calyx lobes that are not reflexed in fruit, style exserted beyond the anther tube for more than 1 mm, stigma globose and capitate, and fruit with the surface not markedly shiny.

#### Type.

**Peru:** Cajamarca: Prov. Cajabamba, in town of Cajabamba, 7°36'43"S, 78°03'28"W, 2649 m, 9 May 2013 (fl, fr), *S. Knapp, T. Särkinen, H.M. Baden, P. Gonzáles & E. Perales 10575* (holotype: USM!; isotypes: BM!, HUT!, CPUN!).

#### Description.

Herb with woody base, 20–50 cm tall, the individual stems to 1 m long and sprawling. Stems terete or somewhat angled with ridges, pubescent with simple uniseriate 1–4-celled trichomes often clustered along the stem angles; new growth densely pubescent with appressed 1–4-celled simple uniseriate trichomes 0.2–0.8 mm long. Sympodial units difoliate, not geminate. Leaves simple, 4.5–12(–15) cm long, 1.8–8 cm wide, ovate to elliptic; adaxial surface sparsely pubescent with more or less appressed 1-4-celled translucent simple uniseriate trichomes, these denser along the veins; abaxial surface more densely pubescent with simple uniseriate trichomes like those of the upper surface; primary veins 5–8 pairs; base acute and decurrent on the petiole; margins entire or occasionally with shallow lobes in the basal third; apex acute; petiole 0.5–2.5(–5) cm long, occasionally narrowly winged, sparsely pubescent with simple uniseriate trichomes like those of the stems and leaves. Inflorescences lateral and intermodal, 1–2.5 cm long, simple or once-branched, with 3–5(9) flowers, sparsely pubescent with appressed 1–2-celled simple uniseriate trichomes; peduncle 0.4–1.6 cm long, if the inflorescence branched then the peduncle rachis 0.4–0.6 cm long; pedicels 0.6–0.7 cm long, ca. 0.3 mm in diameter at the base and apex, straight and spreading, articulated at the base; pedicel scars spaced ca. 1 mm apart. Buds globose, the corolla only exserted from the calyx tube just before anthesis. Flowers 5-merous, all perfect; calyx tube ca. 1 mm long, the lobes 0.5–0.7 mm long with rounded apices, sparsely pubescent with 1–4-celled translucent simple uniseriate trichomes; corolla 5–6 mm in diameter, stellate, white with a yellow central portion near the base, lobed slightly less than halfway to the base, the lobes ca. 1.5 mm long, 2 mm wide, strongly reflexed at anthesis, later spreading, densely pubescent abaxially with 1–4-celled simple uniseriate trichomes, these usually shorter than the trichomes of the stems and leaves; filament tube minute, pubescent with tangled uniseriate trichomes adaxially; free portion of the filaments ca. 1 mm long, pubescent like the tube; anthers ellipsoid, yellow, ca. 1.5 mm long, 0.7–0.8 mm wide; ovary conical, glabrous; style 3–4 mm long, exserted (0.5)1–2 mm beyond the anther cone, densely pubescent with 2–3-celled simple uniseriate trichomes at the base; stigma globose and capitate, minutely papillate, bright green in live plants. Fruit a globose berry, 4–9 mm in diameter, green at maturity or green and turning purplish black when ripe, the surface not markedly shiny, lacking stone cells aggregates; fruiting pedicels 4–7 mm long, ca. 1 mm in diameter at the base, spreading and becoming somewhat more woody in fruit, usually remaining on the plant after fruit drops; fruiting calyx lobes spreading or appressed to the berry, not reflexed. Seeds 35–45 per berry, 1.2–1.5 mm long, 0.9–1 mm wide, flattened-reniform, yellowish, the surfaces minutely pitted, the testal cells pentagonal in outline.

**Figure 3. F3:**
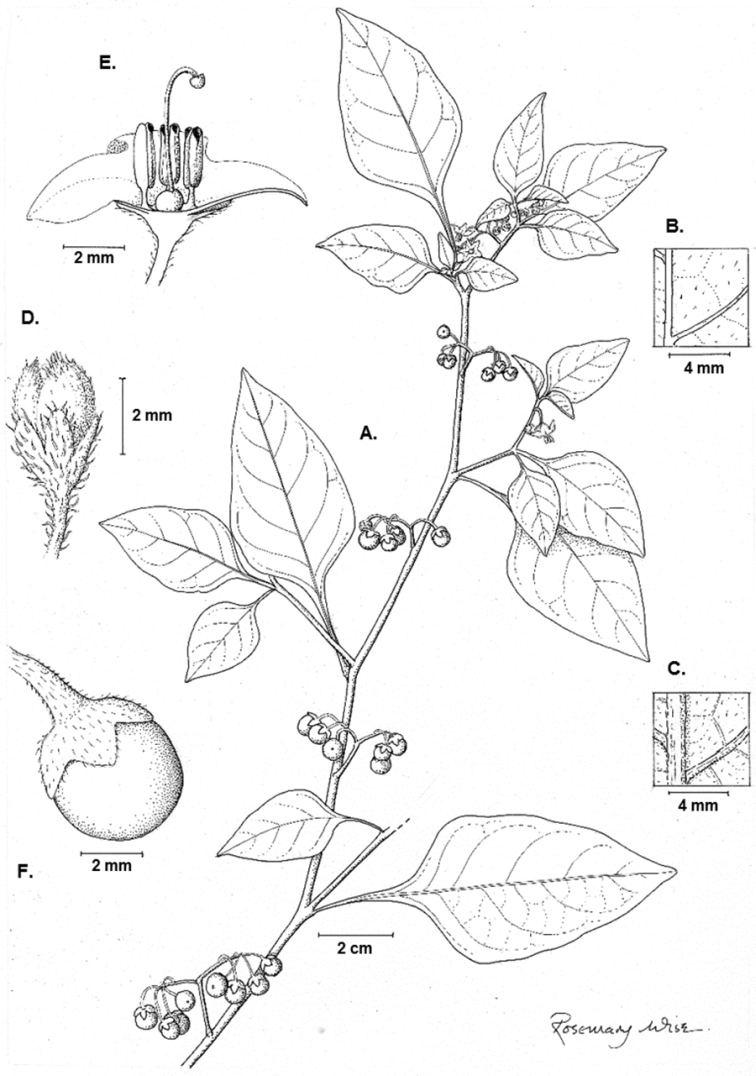
Illustration of *Solanum pseudoamericanum*. A Habit B Adaxial leaf surface **C** Abaxial leaf surface **D** Bud **E** Half flower **F** Fruit (**A–F** *Knapp* 10351). Illustration by Rosemary Wise.

**Figure 4.**
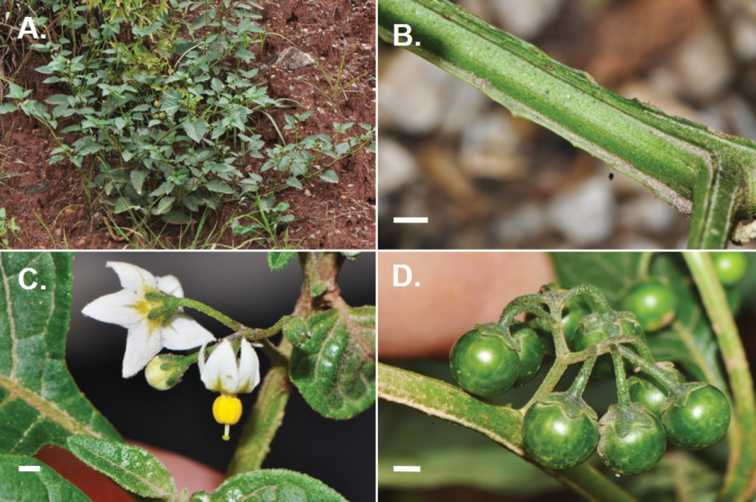
Photos of *Solanum pseudoamericanum*. **A** Habit **B** Ridged stem **C** Flowers with small anthers c. 1.5 mm long, strongly exserted styles and with capitate stigmas **D** Developing fruits which turn purple-black when fully ripe with calyx appressed to the fruit. (**A** *Särkinen et al. 4640*; **B** *Knapp et al. 10357*; **C, D** *Knapp et al. 10300*) Scale bars = 1 mm.

#### Distribution.

Endemic to Peru in the upper zones of seasonally dry tropical forests or in mid-elevation montane forests, usually above 2,000 m elevation, with only some overlap between the closely related *Solanum americanum* that occurs from sea level to 2,200 m in elevation; commonly growing in sandy soils in full sun or partial shade in disturbed sites such as landslides and roadsides or cultivated areas, often in moist depressions in otherwise dry areas, associated with *Schinus molle* L., *Aspidosperma polyneuron* Müll. Arg., *Eriotheca* sp., *Vachellia macracantha* (Humb. & Bonpl.) Seigler & Ebinger, *Alnus acuminata* Kunth, *Solanum probolospermum* Bitter, and *Calceolaria* spp.; (930-)1700–3200(-3735) m in elevation. Based on field and herbarium collections *Solanum pseudoamericanum* occurs in the Departments of Amazonas, Ancash, Apurímac, Cajamarca, Cusco, La Libertad, and Piura, but based on the modelled habitat suitability map (Fig. 2) it is also likely to also occur in the Departments of Lambayeque, Huánuco, Huancavelica, Ayacucho, Junín, southwestern San Martín, northernmost areas of Lima, and in the Province of Loja in Ecuador.

#### Ecology.

Flowering January–July, fruiting March–July.

#### Etymology.

The name *Solanum pseudoamericanum* refers to the fact the new species greatly resembles *Solanum americanum* in general form and has been commonly identified under the name of the more common pantropical weed.

#### Conservation status.

The IUCN threat status of*Solanum pseudoamericanum* is here considered of least concern (LC) based on the relatively large extent of the species occurrence (c. 159,000 km^2^), although the actual area of occupancy is small (96 km^2^). The species grows readily in disturbed sites and combined with the fact that the currently known populations are spread across Peru, it appears to have relatively low threat status despite the generally increasing human pressure and habitat destruction in the Andes.

#### Specimens examined.

**PERU.** **Amazonas**: Chachapoyas, 8 km along road from Leimebamba to Celendín, between km 417–416, 6°42'48"S, 77°49'05"W, 2634 m, 21 Apr 2013 (fl, fr), *T. Särkinen et al. 4624* (USM, BM). **Ancash**: Pallasca, Puente Chucusvalle over Río Tablachaca, left bank of river (other side of bridge in La Libertad), 8°12'10"S, 77°57'06"W, 2148 m, 11 May 2013 (fl, fr), *S. Knapp et al. 10604* (USM, BM); Pallasca, ca. 10 km above Puente Chucusvalle over Río Tablachaca on rd to Pallasca, 8°13'25"S, 77°57'23"W, 2148 m, 11 May 2013 (fl, fr), *S. Knapp et al. 10616* (USM, BM); Huaylas, Dist. Pueblo Libre, just beyond Carapampa village, a few km above bridge over Río Santa, 9°06'28"S, 77°48'42"W, 2637 m, 13 May 2013 (fl, fr), *S. Knapp et al. 10650* (USM, BM); Huaraz, Huaraz, in city, 9°31'51"S, 77°31'27"W, 3003 m, 15 May 2013 (fl, fr), *T. Särkinen et al. 4670* (USM, BM); Carhuaz, on rd from Mancos to Musho, before Puente Apachico, 9°10'35"S, 77°40'31"W, 2886 m, 16 May 2013 (fl, fr), *T. Särkinen et al. 4678* (USM, BM); Corongo, km1-3 on rd to Corongo, a side road from Chimbote-Huaraz main rd, 8°41'38"S, 77°53'51"W, 2334 m, 18 May 2013 (fl, fr), *T. Särkinen et al. 4686* (USM, BM); Pomabamba, just in the outskirts of Pomabamba on rd leading to Piscobamba, 8°49'27"S, 77°27'12"W, 3008 m, 21 May 2013 (fl, fr), *T. Särkinen et al. 4730* (USM, BM); Pomabamba, 2-3km from Pomabamba towards Lucma, 8°51'13"S, 77°26'12"W, 2837 m, 22 May 2013 (fl, fr), *T. Särkinen et al. 4737* (USM, BM); Yungay, ribera del Río, 20 Jul 1977, *Luna, A*., *70* (USM); Carlos F. Fitzcarrald, on rd between Sapcha and San Luis, 9°05'50"S, 77°21'05"W, 3133 m, 24 May 2013 (fl, fr), *T. Särkinen et al. 4778* (USM, BM); Carlos F. Fitzcarrald, in San Luis, outskirts of town, 9°05'35"S, 77°19'42"W, 3147 m, 24 May 2013 (fl, fr), *T. Särkinen et al. 4780* (USM, BM); Huari, c. 5km from Pomachaca on road to Llamellín, 9°23'05"S, 77°06'59"W, 2605 m, 26 May 2013 (fl, fr), *T. Särkinen et al. 4791* (USM, BM); Huari, on rd from Pomachaca to Llamellín, 9°20'24"S, 77°03'22"W, 2571 m, 26 May 2013 (fl, fr), *T. Särkinen et al. 4794* (USM, BM). **Apurímac**: ca. 11 km from Chincheros descending to Río Pampa on Ayacucho-Andahuaylas rd (RN3), 13°31'27"S, 73°46'15"W, 2215 m, 7 Mar 2012 (fl, fr), *S. Knapp et al. 10300* (USM, BM); Chincheros, along Río Pampa on Ayacucho-Andahuaylas rd (RN3), ca. 3-4 km from Puente Pampa on Apurímac side, 13°29'26"S, 73°49'32"W, 2028 m, 7 Mar 2012 (fl, fr), *S. Knapp et al. 10307* (BM,USM); Abancay, village of Tambo, above Curahuasi, on rd from Abancay to Cusco, Dist. Curahuasi, 13°32'21"S, 72°43'02"W, 2673 m, 10 Mar 2012 (fl, fr), *S. Knapp et al. 10351* (USM, BM); Abancay, at turn to Santuario Curahuasi, ca. 17 km above Puente Cunyac over Río Apurímac, road Abancay-Cusco towards Curahuasi, Dist. Curahuasi, 13°32'19"S, 72°29'28"W, 2340 m, 11 Mar 2012 (fl, fr), *S. Knapp et al. 10357* (USM, BM). **Cajamarca**: Cajabamba, in town of Cajabamba, 7°36'43"S, 78°03'28"W, 2649 m, 9 May 2013 (fl, fr), *S. Knapp et al. 10575* (USM, BM); Cajamarca, km1244 on rd from Cajamarca to San Marcos, just outskirts of Namora village, 7°12'04"S, 78°19'33"W, 2764 m, 24 Apr 2013 (fl, fr), *T. Särkinen et al. 4640* (USM, BM). **Cusco**: La Convención, Dist. Echarate, Papelpata, Alto Echarate, 12°46'37"S, 72°36'39"W, 931 m, 24 May 2007 (fl, fr), *G. Calatayud et al. 4062* (NY); Anta, Mollepata, W of Cusco, 13°30'29"S, 72°33'21"W, 3200 m, 10 Jan 1984 (fl), *A.H. Gentry et al. 44135* (MO); La Convención, Santa Teresa, Dist. Santa Teresa, Carretera Santa Teresa-Hidroelectrica, Bosque Seco Secundario, 13°07'21"S, 72°36'31"W, 1700 m, 20 Mar 2004 (fl, fr), *I. Huamantupa et al. 4280* (MO); La Convención, Santa Teresa, Dist. Santa Teresa, Carretera Santa Teresa-Hidroelectrica, Bosque Seco Secundario, 13°07'21"S, 72°36'31"W, 1700 m, 20 Mar 2004 (fl, fr), *I. Huamantupa et al. 4287* (MO,USM). **La Libertad**: Santiago de Chuco, ca. 1 km outside Santiago de Chuco on rd from Shorey and Shorey Chico, at stream crossing, 8°08'38"S, 78°11'08"W, 3735 m, 10 May 2013 (fl, fr), *S. Knapp et al. 10590* (USM, BM); Santiago de Chuco, 6-8 km below Mollepata on rd to river valley of Río Tablachaca, right side of river, 8°12'03"S, 77°57'11"W, 3735 m, 11 May 2013 (fl, fr), *S. Knapp et al. 10599* (USM, BM). **Piura**: Huancabamba, Porculla, km 38, 5°50'25"S, 79°29'38"W, 5°50'25"S, 79°29'38"W, 1800 m, 8 Apr 1989 (fl, fr), *S. Llatas Q. 2348* (NY).

#### Discussion.

Most of the collections of *Solanum pseudoamericanum* are the result of our intensive collecting of Solanaceae in Peru in the last two years. We suspect that the paucity of earlier collections may in part be due to the resemblance to the widespread and weedy *Solanum americanum* that has led to botanists regarding this new species as not worth collecting. Widespread species often harbour cryptic diversity (e.g., Cavers et al. 2013), especially in groups such as the Morelloid clade, where differences between species are relatively small.

*Solanum pseudoamericanum* can be distinguished from the similar *Solanum americanum* by the following suite of characters; berries that are matte or somewhat shiny at maturity, versus very shiny in *Solanum americanum*, styles that are always exerted to approximately equal to the length of the anther cone, versus styles almost included in the anther cone in *Solanum americanum*, and globose, bright green stigmas, versus white or pale green stigmas that are merely a widening of the style tip in *Solanum americanum*. Other members of the Morelloid clade in Peru without glandular trichomes which grow sympatrically with *Solanum pseudoamericanum* differ from it in being larger in growth form reaching up to 2 m in height, having larger, always violet flowers and fruits that are green at maturity (*Solanum probolospermum* Bitter and *Solanum zahlbruckneri* Bitter), or being smaller herbs up to 30 cm high with similar sized flowers but fruits orange or yellow in colour (*Solanum corymbosum* Jacq and *Solanum radicans* L.f.).

## Supplementary Material

XML Treatment for Solanum pseudoamericanum

## Appendix

Occurrence records of *Solanum pseudoamericanum* (doi: 10.3897/phytokeys.31.6312.app) File format: Comma Separated Values (.csv).
